# Clinical Trial Availability by Location for 1000 Simulated AYA Patients

**DOI:** 10.1089/jayao.2021.0014

**Published:** 2022-02-10

**Authors:** Katie L. McLeod, Amber M. Skinner, Lynda K. Beaupin, Susan T. Vadaparampil, Brooke L. Fridley, Damon R. Reed

**Affiliations:** ^1^ Department of Individualized Cancer Management, H. Lee Moffitt Cancer Center and Research Institute, Tampa, Florida, USA.; ^2^ Adolescent and Young Adult Program, H. Lee Moffitt Cancer Center and Research Institute, Tampa, Florida, USA.; ^3^ Cancer and Blood Disorder Institute, Johns Hopkins All Children's Hospital, St. Petersburg, Florida, USA.; ^4^ Department of Health Outcomes and Behavior, H. Lee Moffitt Cancer Center and Research Institute, Tampa, Florida, USA.; ^5^ Office of Community Outreach, Engagement, and Equity, H. Lee Moffitt Cancer Center and Research Institute, Tampa, Florida, USA.; ^6^ Department of Biostatistics and Bioinformatics, H. Lee Moffitt Cancer Center and Research Institute, Tampa, Florida, USA.

**Keywords:** clinical trial, location of care, chemotherapy, treatment navigator, navigation

## Abstract

***Purpose:*** Adolescent and young adult (AYA) oncology patients are less likely to enroll in clinical trials than pediatric patients. After two decades of effort to improve enrollments, challenges remain. We sought to explore where phase II and phase III trials are available for an AYA cohort.

***Methods:*** Based on the epidemiology of AYA cancers and outcomes, we assembled a simulated data set of 1000 patients (AYAsims). Available phase II and phase III trials were matched to diseases and treatment setting (relapsed or newly diagnosed) and characterized by sponsor (industry, National Clinical Trials Network [NCTN], investigator initiated) and location (Moffitt Cancer Center [MCC], community or pediatric).

***Results:*** The majority of AYAsims had potential first line (64.4%) and/or relapsed (68.1%) trials. The majority of these opportunities were industry-sponsored trials available at MCC. Phase II trials for relapsed cancer were most often at the MCC and more likely to be investigator-initiated trials. Trial availability for histologies varied widely, likely reflective of the overall epidemiology of cancers beyond the AYA age range. Pediatric hospitals offered trials for select cancers but had a trial portfolio that matched the fewest number of AYAsims.

***Conclusions:*** In general, newly diagnosed AYA patients have trial enrollment opportunities in both the community and comprehensive cancer center setting with select diagnoses having more trials in pediatric hospitals. Relapsed AYA patients have the most trial opportunities at a comprehensive cancer center. A facile system that navigates patients across health systems would maximize potential AYA trial enrollments.

## Introduction

Adolescent and young adult (AYA) cancer patients, aged 15–39 years, have the lowest participation rates in clinical trials of all cancer patients.^1–5^ This same group comprises 7% of new cancer diagnoses across many different diagnoses and some studies suggest trial enrollment is actually declining.^6^ Clinical trials are the best means to improve outcomes and are often considered the standard of care in cancer.^7^ For example, AYA clinical trial participation has been found to correlate with higher 5-year survival rates in AYA patients with soft tissue and bone sarcomas.^7^ Lack of clinical trial participation in the AYA population also results in less collection of tumor specimens and thus fewer opportunities to learn about disease biology on AYA-specific tumors.^8^ Research and collaborative efforts are necessary to improve AYA trial participation.

While connecting patients to comprehensive cancer centers for care through clinical trial enrollment, the majority of AYAs receive their care in the community oncology setting.^9^ Moffitt Cancer Center (MCC), an NCI-designated comprehensive cancer center located in Tampa, FL, has an established nationally recognized AYA Program that focuses on AYA cancer research and addresses the unique needs of this patient population. In the process of improving collaborations across hospital systems for a multi-institutional grant intended to improve education and subsequent AYA trial enrollment, we encountered a recurrent obstacle: all hospital systems determined the optimal AYA trial portfolio existed within their centers. To address this issue and improve collaboration across hospital systems and settings, we simulated an AYA patient data set and determined which trials were available to them. The goal of this study was to identify gaps in clinical trial enrollment opportunities for AYA patients within our catchment area.

## Methods

### Simulated patient data set

**FIG. 1.**
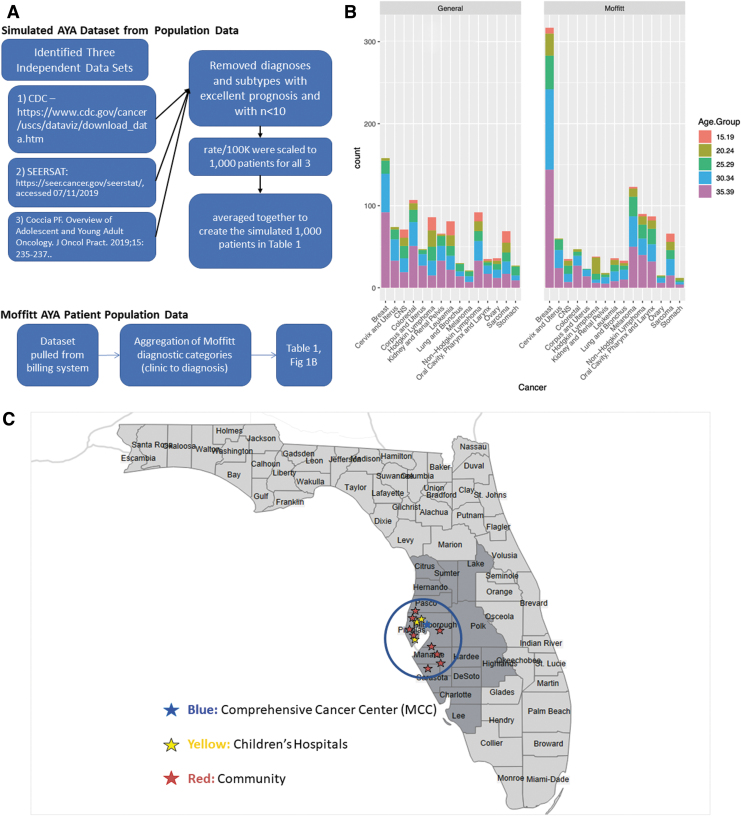

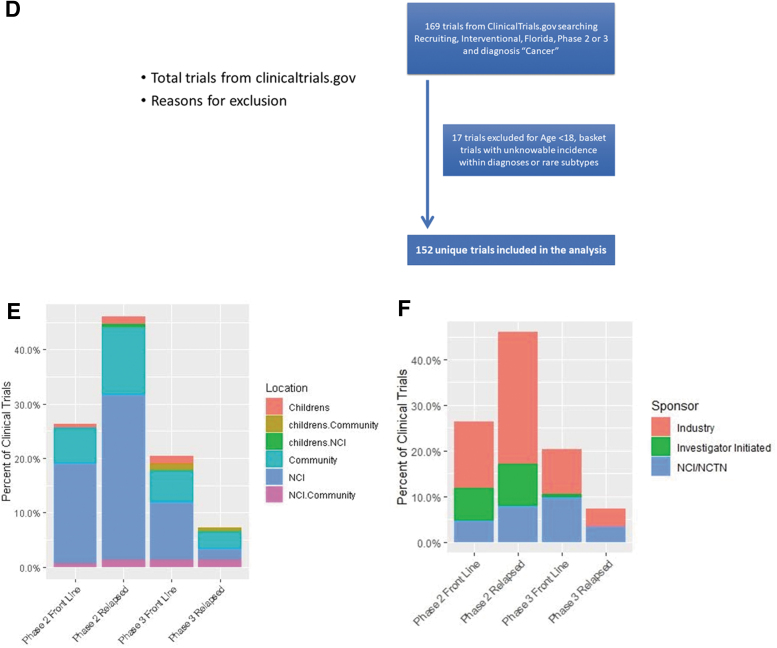
Patient cohort and clinical trials data. **(A)** Creation of simulated AYA data set from epidemiologic data (top) and MCC data set from billing system (bottom). **(B)** Comprehensive cancer center AYA patient data compared with the simulated patient cohort by clinic category with age range. **(C)** Map of MCC catchment area (blue) and available trial locations within 80.45 km from downtown Tampa, FL, denoted by circle. **(D)** Unique phase II and phase III trials available after excluding rare molecular subtypes and basket trials where frequency data within subtypes were either unknown or <2%. **(E)** Unique available AYA phase II and phase III trials categorized by location of trial and **(F)** trial sponsor. AYA, adolescent and young adult; MCC, Moffitt Cancer Center.

We created three independent simulated data sets of 1000 newly diagnosed AYA patients with data generated based on summary statistics from three sources (Fig. 1A).^8,10–12^ Each study had its own category system for diagnoses, but all reported rate per 100,000 and separated by gender into 5-year age range bins. For each data set, the rates per 100,000 for each age group and gender were totaled and scaled to 1000 patients. Any “NA” values in the data were replaced with zeroes. The three data sets were then averaged to create a combined data set of 1000 simulated patients.

### Simulated patient data set exclusions

Cancers with a 5-year survival rate higher than 95% (e.g., thyroid cancer, stages 1 and 2 melanoma, stages 1 and 2 breast cancer, and testicular cancer) were removed from the data set as therapeutic clinical trials are not often conducted for cancers with highly effective frontline therapies.^13^ Diagnoses with <10 simulated patients were also not included, as each only represents <1% of the total population (Fig. 1A). After these exclusions, 16 cancer diagnoses remained (Table 1).

**Table 1.** Simulated 1000 Patient Adolescent and Young Adult Data Set

	15–19		20–24		25–29		30–34		35–39		15–39	%
	Male	Female	Male	Female	Male	Female	Male	Female	Male	Female	Total	
(1) Leukemia	10	7	8	5	5	7	10	7	12	10	81	8.1
(2) Non-Hodgkin lymphoma	7	4	7	5	5	7	14	10	19	14	92	9.2
(3) Hodgkin lymphoma	8	8	10	10	7	10	10	8	8	7	86	8.6
(4) CNS^a^	5	5	5	5	8	7	10	7	11	8	71	7.1
(5) Soft tissue sarcoma	3	3	4	4	5	4	8	5	8	7	51	5.1
(6) Bone sarcoma	5	3	3	1	1	1	1	1	1	1	18	1.8
(7) Melanoma	0	0	0	1	3	3	4	3	4	3	21	2.1
(8) Ovary	0	3	0	4	0	7	0	10	0	12	36	3.6
(9) Corpus	0	0	0	1	0	5	0	14	0	27	47	4.7
(10) Cervix and uterus	0	0	0	3	0	12	0	26	0	33	74	7.4
(11) Breast	0	0	0	3	0	16	0	47	0	92	158	15.8
(12) Colorectal	1	3	4	4	8	7	14	15	27	24	107	10.7
(13) Stomach	0	0	0	1	10	1	3	3	4	5	27	2.7
(14) Kidney and renal pelvis	0	1	1	1	8	4	10	8	19	14	66	6.6
(15) Oral cavity, pharynx, and larynx	1	1	1	1	1	3	5	5	10	7	35	3.5
(16) Lung and bronchus	0	0	0	1	8	1	3	3	7	7	30	3.0
Total	40	38	43	50	69	95	92	172	130	271	1000	

^a^ Central nervous system lymphoma.

### Simulated patient data set subtyping

Diagnoses were split into subtype when available and when it would affect trial matching. Hodgkin lymphoma, central nervous system (CNS) tumors, ovarian, and corpus cancers were not subtyped as there was no subtyping information available. The remaining 12 diagnoses were characterized by stage and subtype when applicable. Surveillance, Epidemiology and End Results data were used for all subtyping, with additional supplementation when needed in the case of breast cancer that was subtyped by hormone receptor and HER2 status and then further by stage using an AYA-specific publication.^14^

### Comprehensive cancer center patient data (MCC data set)

Data for new AYA patients seen at MCC were collected and categorized by disease type. Soarian, an all-inclusive clinical workflow tool, was used to populate AYA patient billing data from July 1, 2018, to June 30, 2019, and then analyzed to determine the appropriate diagnosis code totaling 1593 AYA new patients with diagnostic information (Cerner, Kansas City, Missouri). To mirror the simulated patient data set, diagnoses with excellent prognoses (e.g., thyroid), making up <1% of cases (e.g., myelodysplastic syndrome, multiple myeloma, and pancreatic cancer), or that could not be accurately characterized (i.e., diagnosis category of “other”) were excluded. An additional category of patients without diagnostic information was excluded that was the only difference between the MCC and simulated patient data set. The remaining diagnoses were then normalized to a 1000 patient data set to match the rates observed in the simulated patient data set. Aggregation of diagnostic categories was done to match the more general epidemiologic categories represented in the simulated data set. Sixteen categories resulted, as sarcoma could not be split by bone and soft tissue in the MCC data set. The full data set is given Supplementary Table S1.

### Available clinical trials

Clinical trial information was collected from clinicaltrials.gov from August 26, 2019, to December 18, 2019. Data searches for each subtype were filtered by selecting the following filters: (1) List Tab: status/recruiting, study type/interventional (clinical trial), study phase (II or III); (2) Map tab: Florida. Trial sites that were >80.45 km from Tampa were manually excluded. This was inclusive of the largest community early phase center and three children's oncology group sites. For each histologic subtype, the trials were categorized as phase II or III and either for the newly diagnosed population (front line) or for the same set of patients in relapse (second line and beyond). Phase I trials were not included. Trial inclusion and exclusion criteria were reviewed and trials were matched to simulated patients by stage using “advanced” and “metastatic” for higher stage. Trials evaluating interventions to ameliorate side effects or nontherapeutic trials were also excluded. Trials enrolling for a molecular marker that were agnostic to histology were excluded, as incidence information across cancer types is often unknown or unverified, especially in the AYA population.

## Results

### Simulated patient data set

The simulated new number of cases, AYAsims, across oncologic diagnoses, after excluding both very rare subtypes and staged histologies with an excellent prognosis of >95% 5-year survival, resulted in 16 diagnostic categories (Fig. 1A and Table 1). More granular data are available (Supplementary Table 1). In addition, we compared the actual new patient data by clinic category at MCC with the simulated patient data set in Supplementary Table S2 and Fig. 1B. The data set of 1000 AYAsims, comprising 16 different cancer types, was used for all subsequent analyses.

### Clinical trial characteristics

A total of 152 unique trials were identified (Fig. 1D). The majority of available trials were phase II trials (72.4%). The majority of trials were available at MCC at 101 (66.4%), followed by 53 at community oncology practices, and 9 at pediatric hospitals (Fig. 1E). Only 15 (10%) trials were open at more than one location. The majority, 87 (57%), of trials were industry sponsored, 29 (26%) were National Clinical Trials Network (NCTN) studies, and 26 (17.1%) were investigator initiated (Fig. 1F). Twenty-four trials included multiple diagnoses with the majority being immune checkpoint combination studies.

### Frontline trials

**FIG. 2.**
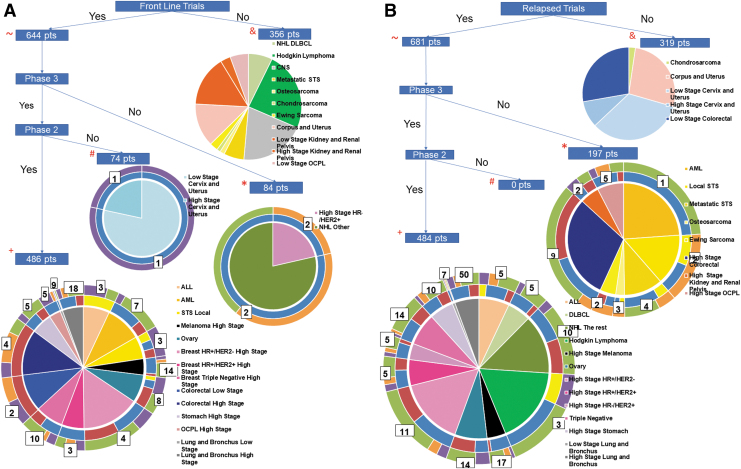
Flow diagram of trial availability for the 1000 patient simulated cohort. **(A)** Frontline trial availability. ^∼^Frontline trials were available for 644 of the 1000 AYA patients. ^&^356 individuals ineligible for frontline trials, ^#^74 were eligible for phase III trials only, ^+^486 were eligible for both phase II and phase III trials, and *84 patients were eligible for phase II trial only. The center circle of each pie chart represents the cancer subtypes. The middle ring displays the trial locations aligning within each cancer subtype; yellow represents pediatric, blue represents MCC, and red represents community hospitals. The number between the middle and outer ring is the number of available trials for the specific diagnosis. The outer ring displays the relative number of trial sponsors; purple represents NCTN, orange represents institutions, and green represents industry. **(B)** Relapsed trial availability. ^∼^Relapsed trials were available for 681 of the 1000 AYA patients. ^&^319 individuals were ineligible for frontline trials, ^#^there were no phase III relapsed trials available for AYAsims, ^+^484 were eligible for both phase II and phase III trials, and *197 patients were eligible for phase II trial only. The center circle of each pie chart represents the cancer subtypes. The middle ring displays the trial locations aligning within each cancer subtype (same color codes as frontline). The number between the middle and outer ring is the number of total trials available for the specific diagnosis. The outer ring displays the relative number of trial sponsors (same color codes as frontline). NCTN, National Clinical Trials Network.

A total of 101 unique frontline trials were available for 644 of the 1000 AYAsims (Fig. 2A). For newly diagnosed AYAsims, the majority of frontline trials were at MCC 63 (62.4%), followed by 33 in the community, and 5 at pediatric hospitals. Of the 644 with an available trial, 74 AYAsims had phase III trials only and 84 had access to phase II trials, with most investigator initiated and located at MCC. The diagnoses of the 356 AYAsims without an available trial are shown (Fig. 2A).

The remaining 486 AYAsims had access to 87 different phase II and phase III trials. Industry sponsored the majority 47 (54%) of these trials, followed by 27 NCTN sponsored trials and 13 investigator-initiated trials. By location, 57 (66%) were at MCC, 33 at community sites, and 5 at pediatric centers. Eight of the 16 different cancer types had both phase II and phase III trials, including breast (HR+/HER2- high stage and triple negative high stage), colorectal, leukemia, lung, melanoma, oral cavity/pharynx and larynx, sarcoma, and stomach cancer.

### Relapsed trials

Trials were available for 681 of the same 1000 AYAsims in relapse (Fig. 2B). Of the 681 simulated patients who had available relapsed trials, 197 patients had access to 24 unique phase II trials only. The majority, 14 (58%), were industry sponsored, 8 investigator initiated, and 2 by NCTN. For relapsed AYAsims, 484 patients had a total of 156 unique phase II and phase III trials available with 98 (63%) of these trials being located at MCC, 56 (36%) at community locations, and 2 (1%) at pediatric locations. Again, the majority were industry sponsored (66%), whereas 19% were NCTN and 15% were investigator initiated.

### All AYA trial characteristics by location and subtype

**FIG. 3.**
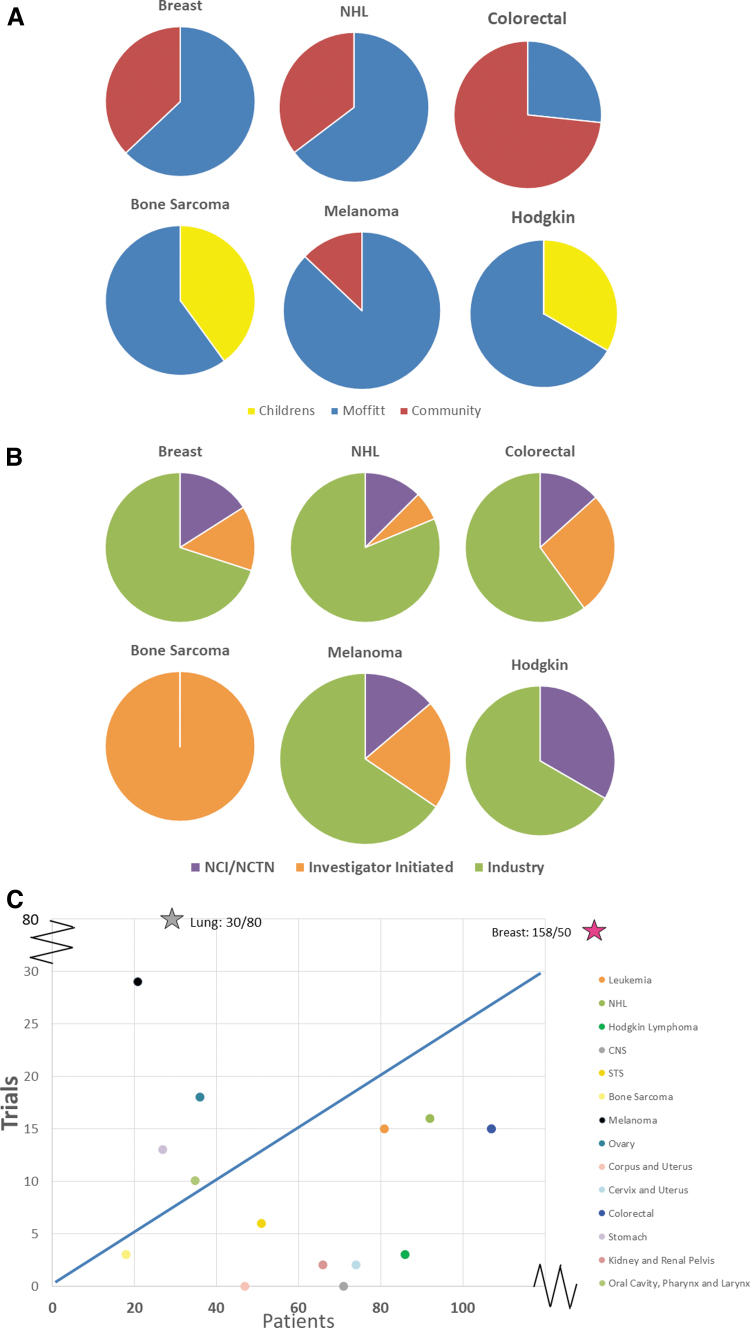
Trial characteristics by cancer type. **(A)** Available trials by location for selected histologies. **(B)** Available trials by sponsor for selected AYA cancer diagnosis. **(C)** Patient number by available trials per diagnosis. The blue line demarcates a trial available for four AYAsims and both breast carcinoma and lung carcinomas are outliers.

By location, 80% AYAsims had either frontline or relapsed trials available at MCC, 22% at pediatric hospitals, and 53% had trials at community centers. The four largest cohorts of cancer subtypes were breast cancer (15.8%), colorectal cancer (10.7%), non-Hodgkin lymphoma (9.2%), and Hodgkin lymphoma (8.6%) (Table 1). Of these diagnoses, 51 of 89 unique trials (57%) were at MCC, 37 in the community, and 1 at pediatric hospitals. For these 4 diagnoses, 59 (70%) of trials were industry sponsored, 13 through the NCTN groups, and 12 investigator initiated (Fig. 3A). Representative diagnoses with the fewest patients included bone sarcoma and melanoma (Table 1). MCC had the highest number of trials available for these diagnoses with 30 of 36 (83%), followed by 4 in the community and 2 at pediatric hospitals. (Fig. 3B). Industry, again, sponsored the majority of these trials, 19 of 32 (59%, 4 trials at multiple sites), followed by 9 investigator initiated and 4 NCTN trials. Available trials by diagnosis was plotted, demonstrating varying ratios of trials per AYAsims by diagnosis, and notably there was an abundance of lung cancer trials and relative few trials for several diagnoses (Fig. 3C).

## Discussion

We found that the majority (*n* = 803/1000) of AYAsims in a 50-mile radius of a comprehensive cancer center had an available trial in either the frontline (*n* = 644/1000) and/or relapse (681/1000) setting. These numbers clearly are much higher than AYA trial enrollment rates and thus we focus on our methodology and both potential future directions from similar works and limitations from the decisions we made in the context of AYA trial enrollment literature. We created a representative data set of 1000 AYAsims most likely to be in need of a clinical trial by removing those with an excellent prognosis and eliminating very rare cohorts. These results thus overestimate the trials available for AYA patients overall. We only included available phase II and phase III interventional clinical trials and characterized them according to location (MCC, community setting, or pediatric hospital), sponsor (industry, NCTN, investigator initiated), and whether they were for newly diagnosed or relapsed patients. Overall, phase III trials were almost evenly split between NCTN and industry sponsorship, whereas the majority of phase II trials were industry sponsored. In addition, the analyses include the same representative data set created for both newly diagnosed and relapsed AYA patients. Thus, we do not capture the true incidence of relapsed populations based on disease-specific event-free survival rates. Similarly, we did not include biomarker-specific basket trials where incidence within a population was low or unknown nor phase I trials.

MCC provided 66% of the total clinical trials covering the majority of patients across the AYA population either newly diagnosed or in relapse. The 6% of the total trials available at pediatric centers were logically for cancers seen in the younger AYA age range: leukemia, soft tissue sarcomas, bone sarcomas, ovarian cancer, and Hodgkin's lymphoma. The community hospitals had 35% of the available trials available. We acknowledge that the results of a similar analysis could have different results with differing variables such as including more or less AYA diagnoses by diagnosis type or prognosis, changing the era of trials, or including phase I or basket trials. These findings are not static, especially when potentially practice changing phase III studies are ongoing. By histology, the available trials, sponsor, and location vary widely. For example, lung carcinomas and melanoma had more available trials than AYAsims during our interval of data collection that is likely reflective of the diagnosis being more common in older adults, having a relatively poor outcome with standard agents, and the current investigational landscape of both promising checkpoint inhibitor and targeted therapies in this cancer type that are demonstrating survival improvements. Other areas (e.g., CNS and uterine carcinomas) have a relative paucity of trials for the AYAsims. Recent literature indicates that within the past decade, improvements have been made in clinical trial enrollment among the older adolescent (younger AYA) cancer population and AYAs with acute lymphoblastic leukemia due to a concerted effort of pediatric and adult cooperative groups to be inclusive of age groups across trials.^1,6^ Furthermore, compared with other age groups within AYAs, those between 15–19 years of age are more likely to be treated at a pediatric center where clinical trials are offered versus older AYAs seeking treatment at nonacademic centers without clinical trial availability.^1^

To help address the issue of older AYAs seeking treatment in the community setting, the National Community Oncology Research Program (NCORP), formerly the Community Clinical Oncology Program (CCOP), was established to increase access to NCI-sponsored clinical trials at community-based cancer treatment centers. However, when examining the enrollment of AYAs onto NCI studies at participating NCORP centers, a significantly lower proportion of AYAs was enrolled at CCOP compared with non-CCOP sites.^9^ And more recently, when comparing AYA enrollment onto Southwest Oncology Group (SWOG) trials, CCOP centers had decreased enrollment than at comprehensive cancer centers, and a decline in AYA age groups across all centers.^15^ Ongoing efforts are needed to evaluate how to improve AYA trial enrollment across centers. The NCTN may be a positive contributing factor in the years to come. In 2014, the National Cancer Institute reorganized the cooperative group structure and established the NCTN. The NCTN reduced the number of adult patient cooperative groups from 10 to 4 and included the children's oncology group. In theory, NCTN efforts enhance pediatric and medical oncology collaboration early on in clinical trial development and offer opportunities to cosponsor trials and hopefully improve AYA enrollment.

But having trials available and enrolling AYA patients on trials are not seamless. There is an excellent body of recent literature investigating barriers to AYA clinical trial enrollment, and we use the framework of Siembida et al. for context of our scope of work and limitations.^16^ In this framework, available trial, accessibility, eligibility, presentation as option, and acceptance are the steps to a successful enrollment. Our findings illustrate trial availability for specific diagnoses and the 50-mile radius enhances the accessibility at least from a logistic standpoint. We could not investigate insurance contracts and other factors affecting an individual AYAsim's location of care. Although not specifically addressing eligibility, we find it uncommon for AYAs to be ineligible for trials due to their relative health and exclusion criteria that typically filter diseases of age such as heart failure. Our methodology did not capture physician awareness or presentation of trials and we address this barrier in future steps hereunder.

In recent years, the NCI placed substantial focus on ensuring that NCI-designated comprehensive centers are serving the unique needs of the community in which they are located.^17^ As such, centers must define, justify, and demonstrate impact within a geographic region (i.e., the catchment area), typically at the level of state, county, or zip code boundaries.^18^ Part of this focus rests on ensuring appropriate availability and access to therapeutic clinical trials for all patients within the catchment area (not only those seen by the center) to address the barrier of limited access, which can be caused by several factors including but not limited to travel limitations, financial toxicity effects of cancer treatment, and other long-term effects and higher uninsured rates. By offering trials that reflect the cancer burden of patients from race/ethnicity, gender, and age groups and addressing the factors causing limited access can influence AYA patient enrollment.

The distribution of clinical trials for AYAs among different hospital settings is due to the variation of cancer types seen across AYA age groups. Although strategies include internal improvements within MCC (e.g., trial navigation, examining trial portfolio to align with AYA cancer burden), greater impact will be achieved by also considering strategies to address external barriers.^16^ As shown by findings in the current project, while the majority of trials were available at MCC, a subset were only available in other settings. Thus, bidirectional partnerships that reduce duplication of effort to open certain trials in more than one location and enhance provider's perception of availability of applicable clinical trials can improve AYA trial enrollment. With unique trials being available in different settings for multiple diagnoses, a system that seamlessly transfers patients across hospital systems would be needed to maximize trial enrollment in both newly diagnosed and relapsed patients. We thus cannot conclude that there is a single best location for all AYA patients to maximize AYA trial enrollments even as we add more evidence that comprehensive cancer centers provide the best probability of AYA trial enrollment. Additional similar investigations in other regions of the country can greatly increase the generalizability of this investigation. A focus with the same methodologies for rarer malignancies that affect the AYA population may also be a next direction. This methodology may assist emerging and existing AYA programs and research toward addressing the infrastructure needs, including communication between medical centers, to improve the rates of trial accrual and outcomes for AYA patients.

## Supplementary Material

Supplemental data
