# Supplementary material for: Clinical Trial Availability by Location for 1000 Simulated AYA Patients
Source: J Adolesc Young Adult Oncol. 2022 Feb 10;11(1):95–103. doi: 10.1089/jayao.2021.0014 (PMC8864422; doi:10.1089/jayao.2021.0014)
Supplement: Supplemental data [file Supp_Table1-2.docx]

**Table 1: Simulated 1,000 patient AYA Data set**

|  |  | **15-19** | | **20-24** | | **25-29** | | **30-34** | | **35-39** | | **15-39** | **%** |
| --- | --- | --- | --- | --- | --- | --- | --- | --- | --- | --- | --- | --- | --- |
|  |  | **Male** | **Female** | **M** | **F** | **M** | **F** | **M** | **F** | **M** | **F** | **Total** |  |
| **1** | **Leukemia** | **10** | **7** | **8** | **5** | **5** | **7** | **10** | **7** | **12** | **10** | **81** | **8.1%** |
| **2** | **Non-Hodgkin Lymphoma** | **7** | **4** | **7** | **5** | **5** | **7** | **14** | **10** | **19** | **14** | **92** | **9.2%** |
| **3** | **Hodgkin Lymphoma** | **8** | **8** | **10** | **10** | **7** | **10** | **10** | **8** | **8** | **7** | **86** | **8.6%** |
| **4** | **CNS^[[1]](#footnote-1)^** | **5** | **5** | **5** | **5** | **8** | **7** | **10** | **7** | **11** | **8** | **71** | **7.1%** |
| **5** | **Soft Tissue Sarcoma** | **3** | **3** | **4** | **4** | **5** | **4** | **8** | **5** | **8** | **7** | **51** | **5.1%** |
| **6** | **Bone Sarcoma** | **5** | **3** | **3** | **1** | **1** | **1** | **1** | **1** | **1** | **1** | **18** | **1.8%** |
| **7** | **Melanoma** | **0** | **0** | **0** | **1** | **3** | **3** | **4** | **3** | **4** | **3** | **21** | **2.1%** |
| **8** | **Ovary** | **0** | **3** | **0** | **4** | **0** | **7** | **0** | **10** | **0** | **12** | **36** | **3.6%** |
| **9** | **Corpus** | **0** | **0** | **0** | **1** | **0** | **5** | **0** | **14** | **0** | **27** | **47** | **4.7%** |
| **10** | **Cervix and Uterus** | **0** | **0** | **0** | **3** | **0** | **12** | **0** | **26** | **0** | **33** | **74** | **7.4%** |
| **11** | **Breast** | **0** | **0** | **0** | **3** | **0** | **16** | **0** | **47** | **0** | **92** | **158** | **15.8%** |
| **12** | **Colorectal** | **1** | **3** | **4** | **4** | **8** | **7** | **14** | **15** | **27** | **24** | **107** | **10.7%** |
| **13** | **Stomach** | **0** | **0** | **0** | **1** | **10** | **1** | **3** | **3** | **4** | **5** | **27** | **2.7%** |
| **14** | **Kidney and Renal Pelvis** | **0** | **1** | **1** | **1** | **8** | **4** | **10** | **8** | **19** | **14** | **66** | **6.6%** |
| **15** | **Oral Cavity, Pharynx and Larynx** | **1** | **1** | **1** | **1** | **1** | **3** | **5** | **5** | **10** | **7** | **35** | **3.5%** |
| **16** | **Lung and Bronchus** | **0** | **0** | **0** | **1** | **8** | **1** | **3** | **3** | **7** | **7** | **30** | **3.0%** |
|  | Total | **40** | **38** | **43** | **50** | **69** | **95** | **92** | **172** | **130** | **271** | **1,000** |  |

**Supplemental Table 1: AYAsims, additional diagnostic detail**

|  |  | **15-19** |  | **20-24** |  | **25-29** |  | **30-34** |  | **35-39** |  | **Total** |
| --- | --- | --- | --- | --- | --- | --- | --- | --- | --- | --- | --- | --- |
|  |  | **Male** | **Female** | **Male** | **Female** | **Male** | **Female** | **Male** | **Female** | **Male** | **Female** | **Diagnosis** |
| **1** | **Leukemia** | **10** | **7** | **8** | **5** | **5** | **7** | **10** | **7** | **12** | **10** | **81** |
|  | **ALL^[[2]](#footnote-2)^** | **7** | **4** | **4** | **2** | **2** | **3** | **4** | **1** | **4** | **3** |  |
|  | **AML^[[3]](#footnote-3)^** | **3** | **3** | **4** | **3** | **3** | **4** | **6** | **6** | **8** | **7** |  |
| **2** | **Non-Hodgkin Lymphoma** | **7** | **4** | **7** | **5** | **5** | **7** | **14** | **10** | **19** | **14** | **92** |
|  | **DLBCL^[[4]](#footnote-4)^** | **1** | **1** | **3** | **1** | **1** | **3** | **4** | **3** | **5** | **4** |  |
|  | **Non-DLBCL** | **6** | **3** | **4** | **4** | **4** | **4** | **10** | **7** | **14** | **10** |  |
| **3** | **Hodgkin Lymphoma** | **8** | **8** | **10** | **10** | **7** | **10** | **10** | **8** | **8** | **7** | **86** |
| **4** | **CNS** | **5** | **5** | **5** | **5** | **8** | **7** | **10** | **7** | **11** | **8** | **71** |
| **5** | **Soft Tissue Sarcoma** | **3** | **3** | **4** | **4** | **5** | **4** | **8** | **5** | **8** | **7** | **51** |
|  | **Local** | **1** | **1** | **1** | **3** | **2** | **3** | **5** | **4** | **5** | **4** |  |
|  | **Metastatic** | **2** | **2** | **3** | **1** | **3** | **1** | **3** | **1** | **3** | **3** |  |
| **6** | **Bone Sarcoma** | **5** | **3** | **3** | **1** | **1** | **1** | **1** | **1** | **1** | **1** | **18** |
|  | **Osteosarcoma** | **2** | **1** | **1** | **1** | **0** | **0** | **0** | **0** | **0** | **0** |  |
|  | **Chondrosarcoma** | **0** | **0** | **0** | **0** | **0** | **0** | **1** | **1** | **1** | **1** |  |
|  | **Ewing Sarcoma** | **3** | **2** | **2** | **0** | **1** | **1** | **0** | **0** | **0** | **0** |  |
| **7** | **Melanoma** | **0** | **0** | **0** | **1** | **3** | **3** | **4** | **3** | **4** | **3** | **21** |
|  | **High Stage** | **0** | **0** | **0** | **1** | **3** | **3** | **4** | **3** | **4** | **3** |  |
| **8** | **Ovary** | **0** | **3** | **0** | **4** | **0** | **7** | **0** | **10** | **0** | **12** | **36** |
| **9** | **Corpus** | **0** | **0** | **0** | **1** | **0** | **5** | **0** | **14** | **0** | **27** | **47** |
| **10** | **Cervix and Uterus** | **0** | **0** | **0** | **3** | **0** | **12** | **0** | **26** | **0** | **33** | **74** |
|  | **I** | **0** | **0** | **0** | **1** | **0** | **10** | **0** | **18** | **0** | **23** |  |
|  | **II** | **0** | **0** | **0** | **0** | **0** | **0** | **0** | **3** | **0** | **3** |  |
|  | **III** | **0** | **0** | **0** | **2** | **0** | **1** | **0** | **4** | **0** | **4** |  |
|  | **IV** | **0** | **0** | **0** | **0** | **0** | **1** | **0** | **1** | **0** | **3** |  |
| **11** | **Breast** | **0** | **0** | **0** | **3** | **0** | **16** | **0** | **47** | **0** | **92** | **158** |
|  | **HR+/HER2-** | **0** | **0** | **0** | **1** | **0** | **7** | **0** | **20** | **0** | **47** |  |
|  | **High Stage** | **0** | **0** | **0** | **1** | **0** | **7** | **0** | **20** | **0** | **47** |  |
|  | **HR+/HER2+** | **0** | **0** | **0** | **0** | **0** | **4** | **0** | **10** | **0** | **15** |  |
|  | **High Stage** | **0** | **0** | **0** | **0** | **0** | **4** | **0** | **10** | **0** | **15** |  |
|  | **HR-/HER2+** | **0** | **0** | **0** | **2** | **0** | **1** | **0** | **5** | **0** | **10** |  |
|  | **High Stage** | **0** | **0** | **0** | **2** | **0** | **1** | **0** | **5** | **0** | **10** |  |
|  | **Triple Negative** | **0** | **0** | **0** | **0** | **0** | **4** | **0** | **12** | **0** | **20** |  |
|  | **High Stage** | **0** | **0** | **0** | **0** | **0** | **4** | **0** | **12** | **0** | **20** |  |
| **12** | **Colorectal** | **1** | **3** | **4** | **4** | **8** | **7** | **14** | **15** | **27** | **24** | **107** |
|  | **0** | **0** | **0** | **0** | **0** | **0** | **0** | **0** | **0** | **1** | **1** |  |
|  | **I** | **1** | **3** | **3** | **4** | **1** | **3** | **3** | **4** | **4** | **5** |  |
|  | **II** | **0** | **0** | **0** | **0** | **1** | **1** | **3** | **1** | **5** | **4** |  |
|  | **III** | **0** | **0** | **1** | **0** | **3** | **1** | **4** | **6** | **10** | **8** |  |
|  | **IV** | **0** | **0** | **0** | **0** | **3** | **2** | **4** | **4** | **7** | **6** |  |
| **13** | **Stomach** | **0** | **0** | **0** | **1** | **10** | **1** | **3** | **3** | **4** | **5** | **27** |
|  | **III** | **0** | **0** | **0** | **0** | **0** | **0** | **0** | **0** | **1** | **0** |  |
|  | **IV** | **0** | **0** | **0** | **1** | **10** | **1** | **3** | **3** | **3** | **5** |  |
| **14** | **Kidney and Renal Pelvis** | **0** | **1** | **1** | **1** | **8** | **4** | **10** | **8** | **19** | **14** | **66** |
|  | **I** | **0** | **1** | **1** | **1** | **8** | **3** | **7** | **7** | **14** | **10** |  |
|  | **II** | **0** | **0** | **0** | **0** | **0** | **1** | **0** | **0** | **1** | **1** |  |
|  | **III** | **0** | **0** | **0** | **0** | **0** | **0** | **1** | **1** | **3** | **1** |  |
|  | **IV** | **0** | **0** | **0** | **0** | **0** | **0** | **2** | **0** | **1** | **2** |  |
| **15** | **Oral Cavity, Pharynx and Larynx** | **1** | **1** | **1** | **1** | **1** | **3** | **5** | **5** | **10** | **7** | **35** |
|  | **I** | **0** | **0** | **1** | **1** | **0** | **1** | **3** | **3** | **3** | **4** |  |
|  | **II** | **0** | **0** | **0** | **0** | **0** | **2** | **0** | **1** | **1** | **0** |  |
|  | **III** | **0** | **0** | **0** | **0** | **0** | **0** | **1** | **0** | **2** | **1** |  |
|  | **IV** | **1** | **1** | **0** | **0** | **1** | **0** | **1** | **1** | **4** | **2** |  |
| **16** | **Lung and Bronchus** | **0** | **0** | **0** | **1** | **8** | **1** | **3** | **3** | **7** | **7** | **30** |
|  | **I** | **0** | **0** | **0** | **0** | **0** | **0** | **0** | **1** | **1** | **1** |  |
|  | **II** | **0** | **0** | **0** | **0** | **0** | **0** | **0** | **0** | **0** | **0** |  |
|  | **III** | **0** | **0** | **0** | **0** | **0** | **0** | **0** | **0** | **2** | **2** |  |
|  | **IV** | **0** | **0** | **0** | **1** | **8** | **1** | **3** | **2** | **4** | **4** |  |
|  |  |  |  |  |  |  |  |  |  |  |  | **1000** |

**Supplemental Table 2: MCC 1,000 Patient Data set**

|  |  | **15-19** | | **20-24** | | **25-29** | | **30-34** | | **35-39** | |  | % |
| --- | --- | --- | --- | --- | --- | --- | --- | --- | --- | --- | --- | --- | --- |
|  |  | **Male** | **Female** | **M** | **F** | **M** | **F** | **M** | **F** | **M** | **F** | **Total** |  |
| **1** | **Leukemia** | **1** | **1** | **4** | **2** | **6** | **2** | **6** | **6** | **2** | **6** | **36** | **3.6%** |
| **2** | **Non-Hodgkin Lymphoma** | **2** | **1** | **5** | **5** | **6** | **10** | **7** | **11** | **19** | **19** | **84** | **8.4%** |
| **3** | **Hodgkin Lymphoma** | **1** | **0** | **13** | **6** | **3** | **4** | **2** | **2** | **2** | **4** | **38** | **3.8%** |
| **4** | **CNS** | **2** | **2** | **4** | **2** | **6** | **4** | **6** | **4** | **2** | **5** | **36** | **3.6%** |
| **5** | **Sarcoma** | **7** | **3** | **6** | **4** | **6** | **5** | **7** | **12** | **6** | **8** | **67** | **6.7%** |
| **6** | **Melanoma** | **1** | **2** | **2** | **7** | **6** | **17** | **15** | **20** | **23** | **25** | **119** | **11.9%** |
| **7** | **Testis** | **2** | **0** | **6** | **0** | **7** | **0** | **11** | **0** | **8** | **0** | **35** | **3.5%** |
| **8** | **Ovary** | **0** | **0** | **0** | **1** | **0** | **2** | **0** | **6** | **0** | **6** | **15** | **1.5%** |
| **9** | **Corpus and Uterus** | **0** | **0** | **0** | **0** | **0** | **1** | **0** | **7** | **0** | **13** | **21** | **2.1%** |
| **10** | **Cervix and Uterus** | **0** | **0** | **0** | **1** | **0** | **12** | **0** | **21** | **0** | **23** | **57** | **5.7%** |
| **11** | **Breast** | **0** | **7** | **2** | **24** | **2** | **37** | **1** | **93** | **4** | **134** | **304** | **30.4%** |
| **12** | **Colorectal** | **0** | **0** | **2** | **1** | **2** | **3** | **4** | **7** | **12** | **13** | **44** | **4.4%** |
| **13** | **Stomach** | **0** | **0** | **1** | **2** | **0** | **2** | **2** | **1** | **2** | **2** | **11** | **1.1%** |
| **14** | **Kidney and Renal Pelvis** | **0** | **0** | **2** | **0** | **2** | **1** | **6** | **2** | **1** | **4** | **17** | **1.7%** |
| **15** | **Oral Cavity, Pharynx and Larynx** | **2** | **3** | **1** | **8** | **2** | **16** | **5** | **15** | **6** | **25** | **82** | **8.2%** |
| **16** | **Lung and Bronchus** | **2** | **1** | **2** | **1** | **3** | **2** | **6** | **6** | **5** | **5** | **34** | **3.4%** |
|  | Total by Sex | **19** | **19** | **51** | **63** | **50** | **120** | **80** | **213** | **92** | **292** | **1000** |  |

1. Central nervous system lymphoma [↑](#footnote-ref-1)
2. Acute lymphoctic leukemia [↑](#footnote-ref-2)
3. Acute myeloid leukemia [↑](#footnote-ref-3)
4. Diffuse large B-Cell lymphoma [↑](#footnote-ref-4)
